# ﻿A new species of *Megalaria* (Ramalinaceae, Ascomycota) from Thailand, and recognition of subgenus Catillochroma

**DOI:** 10.3897/mycokeys.93.90962

**Published:** 2022-11-04

**Authors:** Phimpisa Phraphuchamnong, Matthew P. Nelsen, Isabel Distefano, Joel A. Mercado-Diaz, Sittiporn Parnmen, Achariya Rangsiruji, Kawinnat Buaruang, Robert Lücking, H. Thorsten Lumbsch

**Affiliations:** 1 Lichen Research Unit, Department of Biology, Faculty of Science, Ramkhamhaeng University, Ramkhamhaeng, Bangkok, 10240 Thailand Ramkhamhaeng University Bangkok Thailand; 2 Negaunee Integrative Research Center and Grainger Bioinformatics Center, Field Museum of Natural History, 1400 S. DuSable Lake Shore Drive, Chicago, IL, 60605, USA Field Museum of Natural History Chicago United States of America; 3 Committee on Evolutionary Biology, University of Chicago, 1025 E. 57th Street, Chicago, IL 60637, USA University of Chicago Chicago United States of America; 4 Toxicology Center, National Institute of Health, Department of Medical Sciences, Ministry of Public Health, Tivanon Rd., Nonthaburi 11000, Thailand Toxicology Center, National Institute of Health, Department of Medical Sciences, Ministry of Public Health Nonthaburi Thailand; 5 Department of Biology, Faculty of Science, Srinakharinwirot University, Bangkok, 10110 Thailand Srinakharinwirot University Bangkok Thailand; 6 Botanischer Garten und Botanisches Museum, Freie Universität Berlin, Berlin, Germany Freie Universität Berlin Berlin Germany

**Keywords:** Asia, lichens, mangroves, new taxa, tropical diversity

## Abstract

Tropical regions harbor a substantial diversity of lichenized fungi, but face numerous threats to their persistence, often even before previously unknown species have been described and their evolutionary relationships have been elucidated. *Megalaria* (Ramalinaceae) is a lichen-forming genus of fungi that produces crustose thalli, and includes a number of lineages occupying tropical rain forests; however, taxonomic and phylogenetic work on this clade is limited. Here we leverage both morphological and sequence data to describe a new species from the tropics, *M.pachaylenophila*. This taxon forms a crustose thallus, lacks secondary metabolites, and occurs in mangrove forests of Thailand. We supplemented molecular data from this species with data from other species, including two genera related to and occasionally included in *Megalaria*, namely *Catillochroma* and *Lopezaria*. Our analyses revealed *Catillochroma* species form a monophyletic group embedded within *Megalaria*, and we therefore recognize this clade at the subgeneric level. Since we only included the type species of *Lopezaria* in this study, we refrain from proposing a taxonomic conclusion for that clade at the moment. Several taxonomic combinations are made to reflect phylogenetic evidence supporting the inclusion of these species in *Megalaria*.

## ﻿Introduction

Tropical habitats harbor a rich diversity of lichenized fungi, including numerous undescribed or unrecorded taxa (Lücking et al. 2009). The lichen biota of Thailand serves as a prime example of this trend, with the number of known species having more than doubled over the past two decades (Buaruang et al. 2017). Within Thailand, we have recently focused on the lichen biota of mangroves. Coastal forests in the tropics are species-rich (Donato et al. 2011; Friess 2016) but at great risk, with alarming rates of deforestation (Polidoro et al. 2010; Richards and Friess 2016). During our studies of crustose lichens in mangrove habitats of eastern Thailand, the first author collected a species that appeared undescribed and showed affinities with *Megalaria* Hafellner s. lat. In the family Ramalinaceae (Lücking et al. 2017), though, the circumscription and family placement of *Megalaria* have varied among authors.

*Megalaria* was initially circumscribed as a monospecific genus of lichen-forming fungi characterized by the formation of a crustose thallus with biatorine ascomata, a proper exciple and a pigmented epithecium, including only *M.grossa* (Pers. ex Nyl.) Hafellner at the time of its description (Hafellner 1984). Its limits were subsequently expanded to include both newly described species (Ekman and Tønsberg 1996; Fryday 2004b, 2007; Jagadeesh Ram et al. 2007; Lendemer 2007; McCarthy and Elix 2022), as well as species previously placed in *Catillaria* A. Massal. (Ekman and Tønsberg 1996; Fryday 2004a; Galloway 2004) and *Catinaria* Vain. (Schreiner and Hafellner 1992; Nimis 1993; Ekman and Tønsberg 1996). *Megalaria* was initially placed in its own family, Megalariaceae (Hafellner 1984), which was expanded to include the monospecific *Tasmidella* Kantvilas, Hafellner & Elix (Kantvilas et al. 1999). However, molecular data have since demonstrated the placement of *Megalaria* in Ramalinaceae (Ekman 2001; Miadlikowska et al. 2006, 2014; Ekman et al. 2008; Kistenich et al. 2018), while *Tasmidella* was excluded from this family (Kistenich et al. 2018).

Another genus, *Catillochroma* Kalb & Hafellner, was later described for a group of species previously placed in *Lecidea* Ach., *Lecanora* Ach., *Catinaria*, and *Megalaria*, and was distinguished from *Megalaria* on the basis of its bi-layered excipular anatomy, which included an inner layer formed of *textura intricata* with large intercellular spaces usually filled with crystals, and a uniform prosoplectenchymatous outer layer (Kalb 2007). In contrast, the exciple of *Megalaria* was regarded as being uniformly composed of prosoplectenchyma (Kalb 2007). However, historic (Galløe 1929) and modern (Fryday and Lendemer 2010) examinations of the exciple of the type species of *Megalaria*, *M.grossa*, revealed a bi-layered excipular anatomy similar to that of *Catillochroma*, but distinguished by the loose (*Catillochroma*) versus dense (*Megalaria*) spacing of hyphae in the inner layer of *textura intricata* (Fryday and Lendemer 2010). This distinction was further clouded by the discovery of some species, such as *M.beechingii* Lendemer, with intermediate levels of spacing in the layer of *textura intricata* (Lendemer 2007; Fryday and Lendemer 2010). Consequently, excipular anatomy was regarded as insufficient for the segregation of *Catillochroma* from *Megalaria* (Fryday and Lendemer 2010).

In addition to excipular anatomy, *Catillochroma* was also distinguished from *Megalaria* through the presence of zeorin in the thallus; thus all zeorin-producing *Megalaria* species were transferred to *Catillochroma* (Kalb 2007; Lendemer and Knudsen 2008; Fryday and Lendemer 2010). For instance, the development of excipular *textura intricata* in *M.pulverea* was considered intermediate between that of *Megalaria* and *Catillochroma* (Fryday and Lendemer 2010), and its inclusion in *Catillochroma* was based on its synthesis of zeorin (Kalb 2007). However, such segregation based solely on the presence or absence of a single substance was regarded as insufficient (Fryday and Lendemer 2010). The absence of a *masse-axiale* in asci of the type species of *Catillochroma*, *C.endochroma* (Fée) Kalb, and its close relatives, was also suggested as a potential synapomorphy of this group, and it was noted that species in the *C.endochroma* group could potentially be considered distinct from *Megalaria* (Fryday and Lendemer 2010). However, the distinction of this group from *Lopezaria* Kalb & Hafellner was not clearcut (Fryday and Lendemer 2010).

*Lopezaria* was described as a monospecific genus for the tropical and corticolous species *Lopezariaversicolor* (Flot.) Kalb & Hafellner, distinguished mostly by its large ascospores occurring in numbers of two per ascus (Kalb 1990). Similar to *Catillochromaendochroma*, *L.versicolor* also forms a bi-layered exciple with a layer of loosely spaced *textura intricata* (Fryday and Lendemer 2010), and lacks a *masse-axiale* in the tholus (Kalb 1990). In addition, early reports suggested trace amounts of atranorin and zeorin in the thallus of *L.versicolor* (Sipman 1983), while subsequent examinations have failed to detect zeorin (Fryday and Lendemer 2010). *Lopezariaisidiza* (Makhija & Nagarkar) Aptroot & Sipman – the only other species subsequently included in *Lopezaria* (Aptroot et al. 2007) – also lacks both atranorin and zeorin (Makhija and Nagarkar 1981; Sipman 1983; Fryday and Lendemer 2010). Consequently, the distinction between *Lopezaria* from *Catillochroma*, based on the absence of zeorin and synthesis of larger ascospores (Fryday and Lendemer 2010), was regarded as insufficient (Fryday and Lendemer 2010).

Given the challenges of retaining these three genera as distinct, and in the absence of molecular evidence, all species of *Catillochroma* and *Lopezaria* were transferred to *Megalaria* (Fryday and Lendemer 2010). Thus, *Megalaria* was expanded from a monospecific genus restricted to *M.grossa* (Hafellner 1984) to include approximately 48 species globally that typically form apothecia, with some that instead form soredia and lack ascomata (McMullin and Lendemer 2016). Together, this broadly circumscribed *Megalaria* thus encompasses an ecologically broad assemblage of species that are corticolous (Ekman and Tønsberg 1996; Jagadeesh Ram et al. 2007; Fryday and Lendemer 2010; Lendemer et al. 2016; McCarthy and Elix 2016; McMullin and Lendemer 2016), bryophilous, saxicolous and terricolous, and occur in both temperate and tropical habitats (Fryday 2004b, a, 2007; Lendemer 2007; Su and Ren 2017).

Some authors have continued to recognize *Catillochroma* as a distinct genus within Ramalinaceae, instead of adopting a broadly circumscribed *Megalaria* (Lücking et al. 2017; Kalb 2022). Justification for the continued recognition of *Catillochroma* is rooted in the assumption that these species constitute a well-circumscribed group and that sampling for molecular phylogenetic analysis has remained sparse. Most recently, several *Megalaria* species used to justify the dissolution of *Catillochroma* – or described following its synonymization – were transferred from *Megalaria* to *Catillochroma* (Kalb 2022). This included *Megalariayunnanensis*, which was described as being similar to four species, three of which were previously placed in *Catillochroma* (*M.albocincta* [Degel.] Tønsberg, *M.anaglyptica* [Kremp.] Fryday & Lendemer, *M.pulverea*), and one of which (*M.alligatorensis* Lendemer) was described following the synonymization of *Catillochroma* (Lendemer et al. 2016; Wang et al. 2019). These species share excipular features consistent with *Catillochroma* and produce atranorin, zeorin and fumarprotocetraric acid (Kalb 2007; Fryday and Lendemer 2010; Lendemer et al. 2016; Wang et al. 2019). In addition, several other species were transferred, and three species new to science were also placed in *Catillochroma* (Kalb 2022). However, the reciprocal monophyly of *Catillochroma* (and *Lopezaria*) and *Megalaria* remains to be demonstrated with broader molecular sampling.

Here we describe a new species of lichen-forming fungi from mangroves in eastern Thailand, and place it in *Megalaria* on the basis of morphological and DNA sequence data, including new sequences for an additional nine species. While we were unable to obtain sequence data from the type species of *Catillochroma*, our work still permits an evaluation of the phylogenetic relationships of species previously included in the genus *Catillochroma* (Lücking et al. 2017; Kalb 2022).

## ﻿Materials and methods

### ﻿Taxon selection

We sequenced fungal DNA from representatives of the new species, several taxa representing part of *Catillochroma*, as well as additional taxa potentially placed in the broadly circumscribed *Megalaria*. These data were supplemented with publicly-available sequences from additional *Megalaria* taxa (McMullin and Lendemer 2016; Kistenich et al. 2018; Wang et al. 2019), and other members of the broader Ramalinaceae clade G (Kistenich et al. 2018; van den Boom and Alvarado 2019), which includes *Megalaria*. *Biatoravernalis* was selected as the outgroup (Kistenich et al. 2018). Morphological and chemical data were obtained from recent literature and study of the examined material (Ekman and Tønsberg 1996; Kalb 2007, 2022; Lendemer 2007; Fryday and Lendemer 2010; McMullin and Lendemer 2016; Wang et al. 2019; McCarthy and Elix 2022).

### ﻿Molecular methods

DNA was extracted using the Sigma REDExtract-N-Amp Plant PCR Kit (St. Louis, Missouri, U.S.A.) (Avis et al. 2003; Nelsen et al. 2009) and a 20× DNA dilution utilized in subsequent PCR reactions. Portions of the fungal internal transcribed spacer (ITS), mitochondrial small subunit (mtSSU) and nuclear ribosomal large subunit (nuLSU) were amplified using the ITS1F (Gardes and Bruns 1993) and ITS4A (Kroken and Taylor 2001) primers for the ITS, mrSSU1 and mrSSU2R primers (Zoller et al. 1999) for the mtSSU, and the LR0R (Cubeta et al. 1991) and LR3 (Vilgalys and Hester 1990) primers for the nuLSU.

The 12.5 µL PCR reactions consisted of 5 µM of each PCR primer, 0.5 µl diluted DNA, 6.25 µl REDExtract-n-Amp PCR Ready Mix (Sigma-Aldrich, St. Louis, Missouri, U.S.A.), and 0.5–1.5 µL MgCl_2_. The PCR cycling conditions were as follows: 95 °C for 5 min, followed by 35 cycles of 95 °C for 1 min, 53 °C (mtSSU), 60 °C (nuLSU) for 1 min, and 72 °C for 1 min, followed by a single 72 °C final extension for 5–10 min. Samples were visualized on a 1% ethidium bromide-stained agarose gel under UV light and cleaned with ExoSAP-IT Express (Affymetrix Inc, Santa Clara, California, U.S.A.). The 10 µl cycle sequencing reactions consisted of 0.5 µl of Big Dye version 3.1 (Applied Biosystems, Foster City, California, U.S.A.), 3.5 µl of Big Dye buffer, 1–6 µM primer, 1.5 µl of cleaned PCR product and water. Samples were sequenced with PCR primers. The cycle sequencing conditions were as follows: 96 °C for 1 minute, followed by 24 cycles of 96 °C for 10 seconds, 50 °C for 5 seconds and 60 °C for 4 minutes. Samples were precipitated and sequenced in an Applied Biosystems 3730 DNA Analyzer (Foster City, California, U.S.A.). Sequences were assembled in Geneious Prime 2019.2.1 (https://www.geneious.com/), and submitted to GenBank (Table 1).

**Table 1.** Species included in the present study, collection numbers for newly sequenced specimens, GenBank accession numbers for the three loci, and internal DNA numbers for newly sequenced specimens.

Taxon	Collection (Herbarium)	Locality	ITS	mtSSU	nuLSU
*Catillariasuperflua*	Kalb & Elix 35269 (K. Kalb)	Australia, New South Wales	–	OP689726	–
*Catillochromaalleniae*			KX660734	KX660733	–
*Catillochromadanfordianum*	Kalb & Mertens 39720 (K. Kalb)	Australia, Queensland	–	OP689730	–
*Catillochromamareebaense*	Kalb & Mertens 39753 (K. Kalb)	Australia, Queensland	–	OP689728	–
*Catillochromamareebaense*	K. & D. Kalb 40554 (K. & J. Kalb)	Australia, Queensland	–	OP689733	OP689723
*Catillochromaphayapipakianum*	J. & K. Kalb 41927 (K. & J. Kalb)	Thailand, Chiang Mai	OP698025	OP689731	–
*Catillochromaphayapipakianum*	J. & K. Kalb 41762 (K. & J. Kalb)	Thailand, Chiang Rai	OP698026	OP689732	OP689722
*Catillochromaphayapipakianum*	J. & K. Kalb 41877 (K. & J. Kalb)	Thailand, Chiang Mai	–	OP689734	OP689724
*Catillochromapulvereum*			KX660735	–	–
*Catillochromayunnanense*			MK348528	–	–
*Cliomegalariasymmictoides*			MW622003	MW622006	MW621867
*Lopezariaversicolor*			–	AY584622	–
*Lopezariaversicolor*	Mercado-Diaz 1077 (F)	Puerto Rico, Jayuya	–	OP689735	OP689719
*Lopezariaversicolor*	Soto 2174 (F)	Puerto Rico, Jayuya	–	OP689736	OP689721
*Megalariabengalensis*	Kalb 37938 (K. Kalb)	Brazil, Sergipe,	–	OP689729	–
*Megalariacolumbiana*			–	MN508319	–
*Megalariagrossa*			AF282074	MG925883	–
*Megalariagrossa*	Kalb & Jonitz 41079 (K. Kalb)	Ecuador, Azuay	OP698024	OP689727	OP689720
*Megalarialaureri*			AF282075	MG925884	–
*Megalariapachaylenophila*	Phraphuchamnong (RAMK032107)	Thailand, Chumphon province	OP698023	OP689725	OP689718
*Megalariapachaylenophila*	Chum 2024 (RAMK)	Thailand, Chumphon province	OP698020	–	OP689715
*Megalariapachaylenophila*	Chum 2028 (RAMK)	Thailand, Chumphon province	OP698021	–	OP689716
*Megalariapachaylenophila*	Chum 2072 (RAMK)	Thailand, Chumphon province	OP698022	–	OP689717
*Megalaria* sp.	Kalb 38739 (hb. Kalb)	China, Yunnan	OP698027	–	–
*Biatoravernalis*			AF282070	DQ838753	DQ838752
*Nieblahomalea*			MG925987	MG925888	MG926085
*Ramalinasinensis*			MG926018	MG925921	MG926110
*Tylothalliabiformigera*			AF282077	MG925946	MG926129

### ﻿Phylogenetic analyses

Sequences for individual loci were aligned using the G-INS-i algorithm in MAFFT 7.475 (Katoh and Standley 2013) with and the “--leavegappyregion” option. Poorly aligned regions were subsequently re-aligned using the L-INS-i algorithm (MAFFT) and manual refinement in Mesquite (Maddison and Maddison 2021). Ambiguously aligned regions were then removed using GBlocks 0.91b (Castresana 2000) with a minimum block length of 5, a maximum of 10 contiguous non-conserved positions, and the minimum number of sequences required for gaps, flanking and conserved positions was set to half the number of taxa in the alignment. Alignments were concatenated, and a partitioned maximum likelihood (ML) analysis was conducted in RAxML 8.2.12 (Stamatakis 2014). The GTR+G model was applied and each locus was permitted its own parameter estimates. Support was estimated by conducting 1,000 rapid bootstrap pseudoreplicates (Stamatakis et al. 2008). The RAxML analysis was conducted using the CIPRES Science Gateway (Miller et al. 2010). Trait states for taxonomically important characters in this clade were then derived from the literature and plotted on the tips of the phylogeny.

## ﻿Results

**Figure 1.**
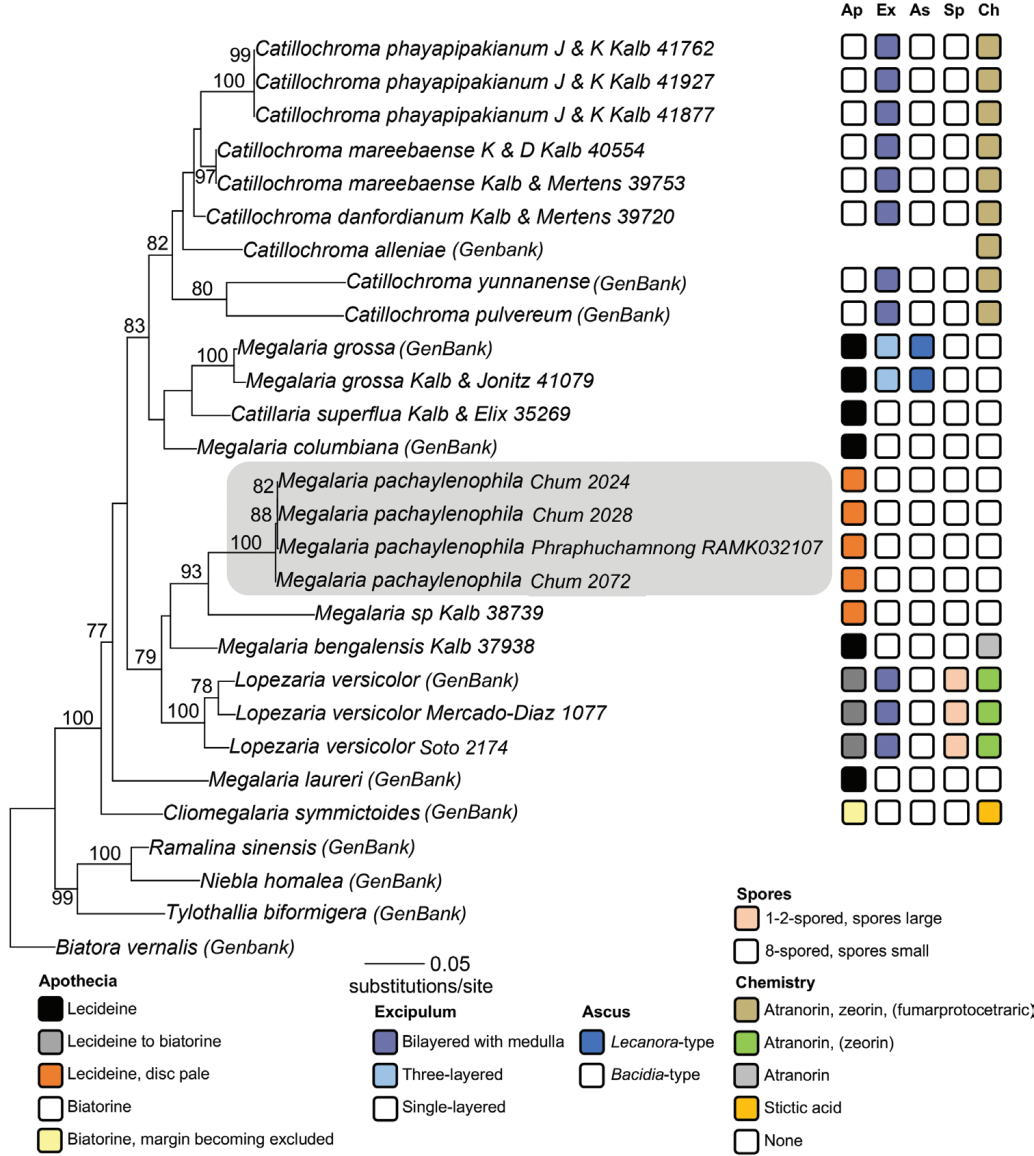
The ITS+mtSSU+nuLSU ML phylogeny with bootstrap values ≥ 70 shown. Newly sequenced specimens include collection info following the species name, while those derived from GenBank are indicated in parentheses. The novel species described here is highlighted in gray. Character states for selected characters are shown at the tips of the phylogeny. Ap = apothecia; Ex = exciple; As = asci; Sp = ascospores; Ch = chemistry.

The final alignment consisted of 1727 characters (ITS: 468; mtSSU: 427; nuLSU: 832). The resulting topology (Fig. 1) revealed good support (bootstrap support ≥ 70) for the monophyly of *Megalaria* s.lat., including the type of *Megalaria* (*M.grossa*), several species of *Catillochroma*, and the type species of *Lopezaria* (*L.versicolor*). The newly discovered species from Thailand was found to be more closely related to *Megalariaversicolor* (the type of *Lopezaria*) than to the type of *Megalaria* (*M.grossa*). Species ascribed to the genus *Catillochroma* formed a strongly supported monophyletic group.

Within the *Catillochroma* clade, *C.pulvereum* (Borr.) Kalb and *C.yunnanense* (C.X. Want & L. Hu) Kalb, two fumarprotocetraric acid-containing species, formed a strongly supported, monophyletic group; however, their relationship to *C.danfordianum* Kalb and *C.phayapipakianum* Kalb – two additional fumarprotocetraric acid-producing species––remains unresolved. Zeorin producing species, which includes the entire *Catillochroma* clade, here represented by *C.yunnanense*, *C.pulvereum*, and *C.alleniae* (Lendemer and McMullin) Kalb, *C.danfordianum*, *C.mareebaense* Kalb and *M.phayapipakianum*, also formed a strongly supported monophyletic group. Species producing atranorin only were paraphyletic including *M.laureri* (Th. Fr.) Hafellner, *L.versicolor*, and *M.bengalensis* Jagadeesh Ram, Aptroot, G.P. Sinha & K.P. Singh. The new species described here lacks substances entirely, and was embedded in a clade that includes atranorin producing species. Other sampled taxa deficient in secondary metabolites included *Catillariasuperflua* (Müller Arg.) Zahlbruckner, *Megalariacolumbiana* (G. Merr.) S. Ekman and *M.grossa*.

All species included were corticolous; thus it was not possible to evaluate relationships among corticolous and non-corticolous taxa. While representation was slightly skewed towards the Northern Hemisphere, species included from the Southern Hemisphere (*Catillariasuperflua* [Müller Arg.] Zahlbruckner, *Catillochromadanfordianum*, *C.mareebaense*, *M.bengalensis* and *M.grossa*) did not form a monophyletic group.

## ﻿Discussion

Our study provides the first, albeit limited, insight into the molecular phylogeny of *Megalaria* s. lat. and confirms that species of genera previously recognized as distinct from, or part of, *Megalaria* indeed form a monophyletic group. Sampled *Catillochroma* species were monophyletic, but nested within *Megalaria* s. lat. Hence, recognition of this zeorin-producing clade at the generic level would leave *Megalaria* paraphyletic. The resurrection of *Lopezaria* (and inclusion of the new species) and separation from *Megalaria* would still keep *Megalaria* paraphyletic, and its segregation from *Lopezaria* on the basis of morphological and chemical characters would remain challenging. Hence, we argue for the previously suggested retention of a broadly-defined *Megalaria* that includes both *Catillochroma* and *Lopezaria* (Fryday and Lendemer 2010). Given the monophyly of *Catillochroma* species examined, and the presumed close relationship of the type species to this clade (which was not sequenced here, despite several attempts), we propose to adopt an alternative classification for this morphologically recognizable clade nested with a larger genus. This approach is similar to that adopted in other groups of lichen-forming fungi, such as *Hypotrachyna* (Divakar et al. 2013). The phenotypically recognizable clade *Catillochroma* is below proposed to be recognized at the subgeneric level. This solution avoids creating a paraphyletic *Megalaria*, while also ascribing a taxonomic rank to the synapomorphies observed in species previously classified in *Catillochroma*.

### ﻿Taxonomic novelties

#### ﻿ Megalaria pachaylenophila

Taxon classificationFungiLecanoralesRamalinaceae

Phraphuchamnong, Buaruang & Lumbsch, sp. nov.

9A080850-7B66-5A07-B412-9DD842C7FF42

846158

Fig. 2

##### Type.

Thailand. Chumphon province: Pathio District; Tambon Pak Klong, 10°53.255'N, 99°28.649'E, 5 m elev., mangrove forest, on bark of *Rhizophoramucronata*, 28 March 2019; Kawinnat Buaruang et al., Chum 2771 (RAMK 034555-holotype, F-isotype).

##### Diagnosis.

Similar to *Megalariabengalensis*, but differs in an ochre to brownish apothecial disc (black in *M.bengalensis*) and in lacking isidia and secondary products (atranorin in *M.bengalensis*).

##### Etymology.

The specific epithet refers to the English translation (Pāchāylen) of the Thai name for mangrove (ป่าชายเลน), and philos (greek) = friend, referring to the ecological preference of the new species.

##### Description.

Thallus crustose, corticolous, gray to olive-gray or greenish gray, up to 10 cm in diameter, smooth, cracked, without soredia or isidia. Apothecia biatorine, plain and flat, becoming slightly convex with age, circular in outline or becoming deformed, sessile, 0.3–0.8 mm in diameter; margins black, shining, contrasting strongly with the coloration of the discs; discs beige to brownish, epruinose. Epihymenium 2–5 μm thick, not pigmented or light beige, K–, N–. Hymenium 75–100 μm thick, hyaline, not inspersed. Subhymenium 10–20 μm thick, hyaline. Central hypothecium 50–80 μm thick, pigmented red-brown, K+ wine-red, N–; lateral hypothecium blue to blue-black, K–, N+ purple. Excipulum 15–25 μm thick, comprised of thick, gelatinized hyaline to blue hyphae, not inspersed with crystals, K–, N+ purple. Asci cylindrical to clavate, eight-spored; ascospores narrowly ellipsoid, hyaline, one-septate (rarely simple), thin walled, not halonate, (9–)11–15 × 4–5 μm. Pycnidia not seen.

***Secondary metabolites*.** Thallus K–, C–, and KC–, UV–, no lichen substances found using TLC.

##### Distribution and ecology.

The new species was found in the south-eastern province of Chumphon where it was growing in old mangrove forests on the bark of *Excoecariaagallocha*, *Hibiscustiliaceus*, *Rhizophoraapiculata*, and *Rhizophoramucronata*.

##### Notes.

In the phylogenetic tree, *Megalariapachaylenophila* and *M.bengalensis* cluster together, and indeed, their apothecial anatomy is very similar. However, they can easily be separated by the isidiate thallus in the latter. No other species in *Megalaria* sens. lat. is known to form a beige or brownish apothecial disk. Interestingly, this can be found in some species of Megalaria (Lopezaria) versicolor which is the sister clade to *Megalariapachaylenophila* and *M.bengalensis*. Additional superficially similar species include the North American *M.beechingii*, which differs in having purple-black to jet black apothecia, a margin that is concolorous with disc, and broadly ellipsoid ascospores, that are often kidney bean-shaped (Lendemer 2007). *Catillochromaphayapipakianum*, which was recently described from Thailand (Kalb 2022) and is transferred to *Megalaria* below, is readily distinguished from *M.pachaylenophila* by having larger (16–26 μm long), narrowly ellipsoid to fusiform, ascospores, and containing atranorin, zeorin, and fumarprotocetraric acid.

**Figure 2.**
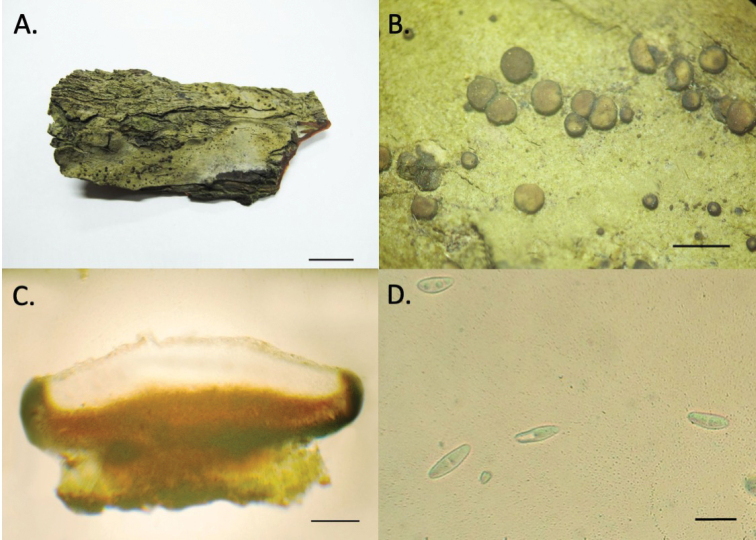
*Megalariapachaylenophila* (RAMK 36122) **A** thallus **B** thallus and ascomata **C** apothecia cross section **D** ascospore. Photos by P. Phraphuchamnong. Scale bars: 1 cm (**A**); 0.5 mm (**B**); 200 µm (**C**); 10 µm (**D**).

##### Additional specimens examined.

Thailand. Chumphon province: Pathio District; Chum Kho sub-district, mangrove forest, on bark of *Rhizophoraapiculata*, 15 Feb 2018; K. Buaruang et al., Chum 2024 (RAMK), 2028 (RAMK), 2072 (RAMK).

Below we propose new combinations to reflect the broad recognition of *Megalaria* and the recognition of the *Catillochroma* clade at subgeneric level:

#### ﻿ Megalaria subgen. Catillochroma

Taxon classificationFungiLecanoralesRamalinaceae

(Kalb) Lücking, Lumbsch & Nelsen, comb. et stat. nov.

C4E42280-F237-57FD-BD75-CA8258DD4389

846159

Catillochroma Kalb, Bibl. Lichenol. 95: 298 (2007). Type species: Catillochromaendochromum (Fée) Kalb.

#### ﻿ Megalaria bicolorata

Taxon classificationFungiLecanoralesRamalinaceae

(Vain.) Lumbsch & Nelsen comb. nov.

C99ABA6C-E38D-5E6A-A5C4-3FCA131E8A9F

846160

Catillochroma bicoloratum (Vain.) Kalb., Archive for Lichenology 30: 12 (2022). – Catillariabicolorata Vain. Annales Botanici Societatis Zoologicae-Botanicae Fennnicae ‘Vanamo’ 1: 48 (1921).

#### ﻿ Megalaria danfordiana

Taxon classificationFungiLecanoralesRamalinaceae

(Kalb) Lumbsch & Nelsen comb. nov.

B42298AF-E92F-5A7D-B450-5F6E9F2A5BC8

846161

Catillochroma danfordianum Kalb., Archive for Lichenology 30: 4–6 (2022).

#### ﻿ Megalaria mareebaensis

Taxon classificationFungiLecanoralesRamalinaceae

(Kalb) Lumbsch & Nelsen comb. nov.

48BE9D13-0455-5D09-B38F-A33A9FC49FB2

846162

Catillochroma mareebaense Kalb., Archive for Lichenology 30: 6–8 (2022).

#### ﻿ Megalaria phayapipakiana

Taxon classificationFungiLecanoralesRamalinaceae

(Kalb) Lumbsch & Nelsen comb. nov.

C6A0D929-0E01-57E3-97AD-C306847F84B3

846163

Catillochroma phayapipakianum Kalb., Archive for Lichenology 30: 8–10 (2022).

#### ﻿ Megalaria superflua

Taxon classificationFungiLecanoralesRamalinaceae

(Müll. Arg.) Kalb, Lumbsch & Nelsen comb. nov.

A38A7C02-29D2-53EA-B538-1C880D255CFF

846164

Catillaria superflua (Müller Arg.) Zahlbruckner., Catalogus Lichenum Universalis 4: 75 (1926). – Patellariasuperflua Müll. Arg., Flora (Regensburg) 70: 336 (1887).

## Supplementary Material

XML Treatment for Megalaria pachaylenophila

XML Treatment for Megalaria subgen. Catillochroma

XML Treatment for Megalaria bicolorata

XML Treatment for Megalaria danfordiana

XML Treatment for Megalaria mareebaensis

XML Treatment for Megalaria phayapipakiana

XML Treatment for Megalaria superflua
